# An Efficient Method for Isolating and Purifying Nuclei from Mice Brain for Single-Molecule Imaging Using High-Speed Atomic Force Microscopy

**DOI:** 10.3390/cells13030279

**Published:** 2024-02-02

**Authors:** Yujia Qiu, Elma Sakinatus Sajidah, Sota Kondo, Shinnosuke Narimatsu, Muhammad Isman Sandira, Yoshiki Higashiguchi, Goro Nishide, Azuma Taoka, Masaharu Hazawa, Yuka Inaba, Hiroshi Inoue, Ayami Matsushima, Yuki Okada, Mitsutoshi Nakada, Toshio Ando, Keesiang Lim, Richard W. Wong

**Affiliations:** 1 Division of Nano Life Science, Graduate School of Frontier Science Initiative, Kanazawa University, Kanazawa 920-1192, Japan; porthos0616@gmail.com (Y.Q.); mi.sandira@unhas.ac.id (M.I.S.);; 2 WPI Nano Life Science Institute (WPI-NanoLSI), Kanazawa University, Kanazawa 920-1192, Japanmhazawa@staff.kanazawa-u.ac.jp (M.H.); tando@staff.kanazawa-u.ac.jp (T.A.); 3 Cell-Bionomics Research Unit, Innovative Integrated Bio-Research Core, Institute for Frontier Science Initiative, Kanazawa University, Kanazawa 920-1192, Japan; 4 Metabolism and Nutrition Research Unit, Institute for Frontier Science Initiative, Kanazawa University, Kanazawa 920-8641, Japan; 5 Laboratory of Structure-Function Biochemistry, Department of Chemistry, Faculty of Science, Kyushu University, Fukuoka 819-0395, Japan; 6 Institute for Quantitative Biosciences, The University of Tokyo, Tokyo 113-0033, Japan; 7 Department of Neurosurgery, Graduate School of Medical Science, Kanazawa University, Kanazawa 920-8641, Japan

**Keywords:** mouse brain, nuclear pore, high-speed atomic force microscopy (HS-AFM), strainer

## Abstract

Nuclear pore complexes (NPCs) on the nuclear membrane surface have a crucial function in controlling the movement of small molecules and macromolecules between the cell nucleus and cytoplasm through their intricate core channel resembling a spiderweb with several layers. Currently, there are few methods available to accurately measure the dynamics of nuclear pores on the nuclear membranes at the nanoscale. The limitation of traditional optical imaging is due to diffraction, which prevents achieving the required resolution for observing a diverse array of organelles and proteins within cells. Super-resolution techniques have effectively addressed this constraint by enabling the observation of subcellular components on the nanoscale. Nevertheless, it is crucial to acknowledge that these methods often need the use of fixed samples. This also raises the question of how closely a static image represents the real intracellular dynamic system. High-speed atomic force microscopy (HS-AFM) is a unique technique used in the field of dynamic structural biology, enabling the study of individual molecules in motion close to their native states. Establishing a reliable and repeatable technique for imaging mammalian tissue at the nanoscale using HS-AFM remains challenging due to inadequate sample preparation. This study presents the rapid strainer microfiltration (RSM) protocol for directly preparing high-quality nuclei from the mouse brain. Subsequently, we promptly utilize HS-AFM real-time imaging and cinematography approaches to record the spatiotemporal of nuclear pore nano-dynamics from the mouse brain.

## 1. Introduction

The nucleus is enclosed by two concentric membranes known as the nuclear envelope (NE). The surface of the NE is covered with hundreds to thousands of minuscule nuclear pore complexes (NPCs) [1,2,3,4,5,6,7,8,9]. NPCs serve as the only gateways between the nucleus and the cytoplasm [10,11,12,13,14,15]. NPCs are tiny gatekeepers that have a central, selective, cobweb-like barrier made up mostly of nucleoporins (Nups) [11,16,17,18,19,20,21]. Nucleoporins are a collection of around 30 distinct proteins that are assembled inside the cell to form biological “nanomachines” that form an eightfold rotationally symmetric core 8 scaffold that can expand between 40 and 60 nm in diameter [10,16,17,18,19,22]. Nucleoporins (Nups) can be classified into three main groups based on their localization and functions: transmembrane Nups, which are integrated into the structure of the nuclear envelope (NE); central scaffold Nups, which provide structural support; and phenylalanine–glycine (FG)-Nups, which constitute the selective barrier within NPCs [4,5,9,12,22,23,24]. Due to the varying anchoring sites of FGs, it is likely that they create distinct FG layers along the pathway via NPCs [25]. Transportation via NPCs is highly specific and rigorous in terms of structural disruptions at the microscopic level and fluctuations in molecular activity [8,26,27]. Small molecules can freely traverse NPCs, while larger molecules (>40 kDa) can only pass smoothly through the pore if they are bound to specific transporter proteins that interact with FG (phenylalanine–glycine)-nucleoporins (FG-Nups) [28,29]. These FG-Nups form a soft and flexible lining consisting of around 200 intrinsically disordered polypeptide (IDP) chains within the turnstile [5,7,16,26,30].

Various models for the trafficking of FG-Nups have been suggested, including the spider cobweb, forest, virtual gating, polymer brush, and selective phase/hydrogel models [19,30]. The malfunctioning of the NPC transport mechanism is linked to a variety of illnesses, including neurodegeneration [31], viral infections [32,33,34,35,36,37], and cancer [38,39].

In addition to nuclear transport, our laboratory investigated other functions of NPCs. We discovered that nucleoporins Rae1, Tpr, Nup358/RanBP2, Nup62, Nup58, and Nup88 are crucial for maintaining spindle bipolarity, centrosome, and mid-body homeostasis during cell division [30]. This phenomenon has since been verified by other groups and is now recognized as a component of the pathway leading to antimitotic catastrophe in early carcinogenesis [30]. We and others have demonstrated the crucial involvement of NPC transcription enhancer proteins in several types of cancer [39,40]. We demonstrated the involvement of the nuclear pore protein Tpr in autophagy a decade ago, a process that has recently had implications for ependymoma [38].

The latest progress in NPC architecture involved integrating state-of-the-art high-resolution cryo-electron microscopy (cryo-EM) [41], advanced artificial intelligence-based deep learning [42,43], and all available structural data obtained from X-ray crystallography, NMR, and mass spectrometry [17]. This resulted in achieving structural modeling with a resolution of less than one nanometer [19,29]. Nevertheless, these studies mostly concentrate on elucidating the arrangement of stationary NPC subcomplexes and do not furnish data regarding the dynamic configuration of the structure, particularly during nuclear transport, which is currently deficient [5].

The advancement of atomic force microscopy (AFM) has revolutionized the investigation of biomolecules [44]. The utilization of AFM has yielded significant results as a result of advancements in precise cantilevers for molecular imaging and ways for modifying cantilevers to quantify single-molecule forces [45]. Traditional atomic force microscopy (AFM) methods, commonly employing *Xenopus* egg nuclear pores, have provided limited information in the form of still images and stiffness measurements at very low speeds [22,25,45,46].

AFM combined with single-molecule localization microscopy (SMLM) shows that the NPC basket has a high degree of flexibility and explores a wide range of conformations. In addition to its typical basket shape, the NPC basket may either penetrate into the central pore channel or expand, with filaments extending toward the edges of the pore. These studies demonstrate the adaptability of this structure, allowing for the transportation of anatomically varied payloads across NPCs [47,48].

However, these methods have not been able to accurately analyze the core channel of FG-Nups due to their insufficient spatial resolution [48]. *Xenopus* oocytes are well suited for studying nucleocytoplasmic transport due to their significantly larger size (1000 μm) compared to mammalian nuclei (10–20 μm). This size difference allows for an easier isolation of NEs [49]. However, it is important to note that there are variations in the composition and functionality of NPCs between different species. Instances of dissimilarity include cell division processes, NE rupture and repair during cancer cell migration, the involvement of oncogenes or tumor suppressors, the nucleocytoplasmic transport of viral components, and chromatin alterations in mammalian cell NPCs, all of which differ significantly from those observed in the NPCs of *Xenopus* eggs. Hence, it is imperative to examine the spatiotemporal dynamics of mammalian NPCs without delay [46].

The scanning speed is a significant challenge when employing conventional AFM to investigate the NPC. In order to address this constraint, researchers have created a customized high-speed atomic force microscope (HS-AFM) specifically designed for use in biological samples [50,51,52,53,54,55,56,57,58,59,60,61,62,63,64,65,66]. The advancement of HS-AFM has resulted in the creation of devices that surpass prior constraints, offering temporal resolution in the sub-second range and spatial resolution in the sub-nanometer range [67,68]. The HS-AFM generally achieves a three-dimensional topographic resolution of 2 to 3 nm in the XY plane and 0.15 nm in the Z direction. HS-AFM employs a nanometer-sharp tip that oscillates fast to occasionally tap on a surface using microsecond pulses (i.e., frequencies in the megahertz range) and forces below 50 piconewtons. This reduces disruptions to a specimen as the impulse or energy being conveyed is insignificantly little [69].

Although cryo-electron microscopy (EM)-derived maps, Alpha Fold, and simulation tools have led to significant progress in refining the structure of the NPC [10,11,26,70,71], the issue of the NPC permeability barrier remains unclear. Through the utilization of HS-AFM, we have demonstrated that FG-Nups exist in a state of liquid–liquid phase separation (LLPS) [72]. This state involves the formation of rapid and temporary structures composed of FG filaments [20], which occasionally coexist with a central plug during conformational changes in cancer cells and organoids [72]. According to the real-time HS-AFM movies, we demonstrated that the extensive intrinsically disordered regions (IDR) of FG-Nups display remarkably dynamic oscillations, resulting in a temporary, continually changing form, suggesting the structure of a radial spider cobweb [20]. The FG domains, along with cargo-carrying transport factors, constitute a structure known as the central plug, which exhibits a high degree of dynamism in mammalian cells and organoids [72].

The utilization of HS-AFM to observe the components of tissues and organs is significantly limited due to the difficulties involved in purifying these components and putting them back on the HS-AFM scanning stage. This restriction mostly applies to culture cells or purified proteins for HS-AFM imaging. Ensuring a dependable and replicable tissue-purifying protocol is crucial for HS-AFM imaging. Here, we present a rapid strainer microfiltration (RSM) procedure for effectively gathering brain nuclei from mice, along with the essential concepts for performing HS-AFM imaging to film the nanotopology of nuclear pore inner filaments.

## 2. Materials and Methods

### 2.1. Nuclei Isolation

Mice brains were harvested and then washed several times with a PBS and NIM1 buffer to remove blood and connective tissues. Brain tissues were minced in an NIM1 buffer and then centrifuged at 300× g for 8 min. The tissue pellet was washed with PBS 1%BSA (300 g, 8 min) and then resuspended in a homogenization buffer (HB). Brain tissues were distributed to several Biomasher II tubes and subjected to centrifugation at 1000 rpm for 2 min prior to homogenization. Tissue pellets were then homogenized for 12–15 strokes using a pestle and then incubated on ice for 10 min. The homogenates were centrifuged at 300 rpm for 5 min. After that, the homogenates were filtered using cell strainers with a descending order of pore sizes. Filtrates were collected and proceeded to centrifugation at 300× g, 10 min. Nuclei pellets were resuspended in PBS 1% BSA and then proceeded to quality control using a fluorescent cell imager (ZOE, Bio-Rad Laboratories, Hercules, CA, USA). Nuclei were either proceeded to immediate HS-AFM scanning or stored at −80 °C with the Bambanker. Impurities found in nuclei suspension can be removed using sucrose density gradient ultracentrifugation.

### 2.2. Immunostaining and Confocal Microscopic Analysis

Nuclei on coverslips were fixed for 10 min in 4% paraformaldehyde (PFA), then permeabilized with 0.3% Triton X-100 in PBS for 10 min at room temperature (RT), and subsequently blocked with a blocking solution (PBS containing 4% BSA and 0.1% Tween 20) for 30 min at room temperature. Coverslips were incubated overnight with the indicated primary antibody (mAB414 (Abcam/ab 24609, Cambridge, UK) 1:1000, lamin B1 (Abcam/ab 16048) 1:1000, Cambridge, UK). Coverslips were washed three times and incubated with Alexa Fluor-conjugated 488 and 555 secondary antibodies (Life Technologies, Carlsbad, CA, USA) for 1 h. After three washes, samples were mounted onto coverslips using the Pro-Long Gold Antifade reagent (Life Technologies, Carlsbad, CA, USA) and DAPI and examined using a fluorescent cell imager (ZOE, Bio-Rad Laboratories, Hercules, CA, USA) and confocal microscopy (Olympus, objective 60× PlanApo/1.45NA, FV10i-LIV, Tokyo, Japan) following the manufacturer’s instructions, respectively.

### 2.3. Electron-Beam Deposition (EBD) for the Nanofabrication of Cantilever Tips

The mechanical probes (cantilevers) utilized were small custom-made cantilevers (BL-AC10DS-KU2, Olympus) with a spring constant (k) of roughly 0.1 N/m and a resonance frequency (f) of 0.6 MHz in water. The dimensions of the cantilever were as follows: a length ranging from 6 to 9 μm, a width of 2 μm, and a thickness of 0.13 μm. Using a field-emission scanning electron microscope (ELS-7500, Elionix, Tokyo, Japan), an elongated, shapeless carbon tip was grown on the apex of each cantilever using electron-beam deposition (EBD). Cantilever cleaning was accomplished using the UV/O3 treatment. The optimization of EBD was achieved by employing a 30 kV accelerating voltage and a 2 min irradiation duration. Utilizing carbon tips that are both sharpened and lengthy (about 3 μm) can enhance the quality of HS-AFM imaging, minimize the squeezing effect, and ensure that oscillation damping is due to the tip–sample contact.

### 2.4. HS-AFM Imaging

The high-speed atomic force microscope was fitted with a wide-range HS-AFM scanner and operated in tapping mode at room temperature, following our earlier reports, with a free amplitude of 2 nm and a frequency of around 900 kHz. We employed EBDed-cantilevers in HS-AFM for the purpose of imaging. The deflection of the cantilever was determined by detecting the location of the laser beam (670 nm) reflected by the cantilever using a position-sensing two-segmented photodiode. The laser beam was directed onto a cantilever tip by means of a 20× objective lens (CFI S Plan Fluor ELWD, Nikon, Tokyo, Japan). The amplitude of the free oscillation was around 2 nanometers, whereas the set-point amplitude was 80–90% of the free amplitude. In order to see delicate mice nuclei, we immobilized nuclei on 0.001% poly-L-lysine-coated mica for 30 min and then washed them with a scanning buffer to remove free nuclei. Finally, nuclei were scanned under a near-physiological buffer (50 mM Tris HCL 150 mM NaCl, pH 7.4).

### 2.5. HS-AFM Image Processing

The HS-AFM videos underwent processing to eliminate the thermal drift in the x, y, and z directions, as well as to diminish high-frequency noise in the x and y directions (specifically, noise originating from the z-scanner). This processing was carried out using Image J software (version 1.53h, Java 1.8) [73]. To begin with, the median filter with a radius of 2.0 was employed to decrease the horizontal noise. Secondly, the z-directional drift was eliminated by subtracting the background from each frame using the principal functions in the x- and y-directions. This process ensured that there was no variation in the average height between frames [20,72].

### 2.6. Analysis of FG-Nups

Ultra-speed was employed to study the FG-Nups network within the central turnstile of the NPC in order to illustrate the activity of this discerning barrier in human cells. The scanning process utilized a fast velocity of 400 ms/frame to cover an area of 120 × 120 nm^2^ within the channel [20,72]. The picture resolution may vary depending on the stability, tip quality, and sample roughness. Subsequent analysis of the initial data was employed to examine the FG-Nups at various time intervals utilizing picture lookup tables. The presence of FG threads was readily discernible.

## 3. Results

### 3.1. Preparation of Mice Brain Nuclei

**Figure 1:**
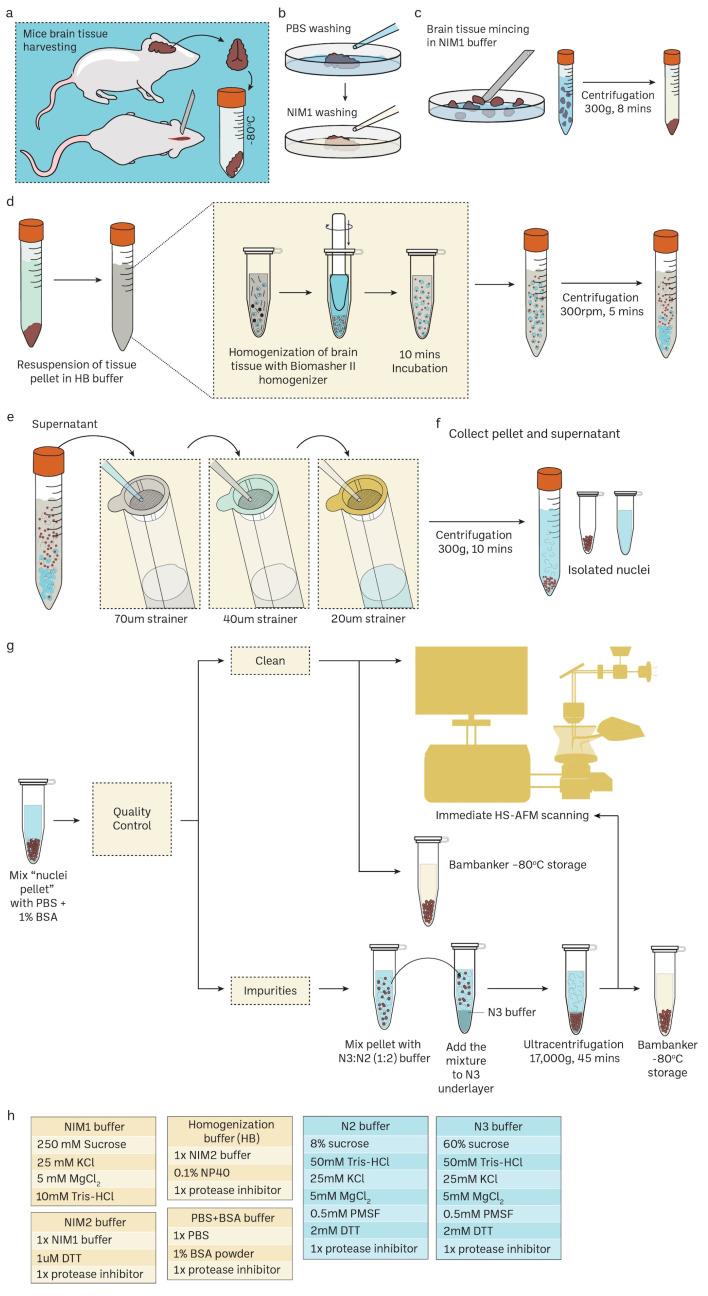
Routine mouse brain nuclei isolation workflow for single-molecule imaging. Detailed protocol in the method Section 2.1 and Supplementary Figure S1. (**a**–**c**) Mice brain harvesting and tissue preparation. (**d**) Mechanical cell rupture to release nuclei using Biomasher II homogenizer. (**e**,**f**) Nuclei isolation using cell strainers with specific pore size in descending order. (**g**) Downstream experiments for isolated nuclei such as confocal imaging and HS-AFM imaging. Sucrose gradient differential centrifugation is an additional step to remove impurities in nuclei samples. Cryoprotectant Bambanker can be used for nuclei storage at –80 °C. (**h**) The recepies of various buffers for mice nuclei isolations.

**Figure 2:**
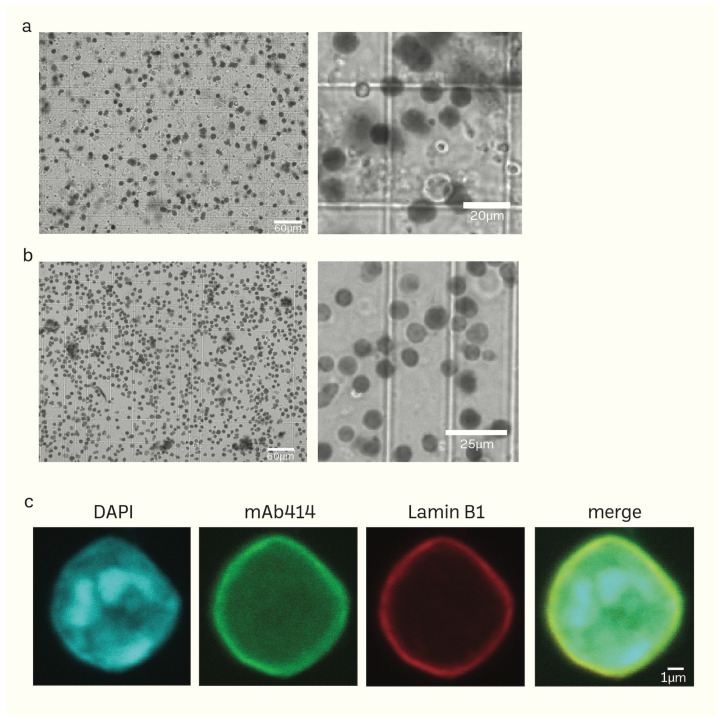
(**a**,**b**) Phase-contrast images of isolated mice brain nuclei captured using the fluorescent cell imager. Impurities together with nuclei (**a**) can be removed using sucrose density gradient centrifugation without damaging the nuclei (**b**). (**c**) Morphology of isolated nuclei of higher resolution epifluorescence images obtained using a confocal microscope (scale bar: 1 μm).

Unlike the sucrose density gradient centrifugation [6,74,75], we have developed a straightforward and effective RSM method for isolating nuclei from mice brain tissue using only a few strainers and a table centrifuge [76]. Figure 1 illustrates the schematic depiction of the research design. The presence of protease inhibitors was ensured throughout each stage to minimize degradation. Nuclei isolation was achieved by douncing and grinding the minced mouse brain tissue with a pestle in the homogenization buffer. The texture of the inner wall of the *Biomasher II* tube and the tip of the pestle enhance the efficiency of homogenization. The homogenization buffer contains low amount NP40, which facilitates the release of nuclei by rupturing the cell membrane. The homogenate was then subjected to a series of filtration using cell strainers in descending order of pore sizes (70 μm → 40 μm → 20 μm). The filtrate was then washed several times with PBS 1% BSA (300 g for 10 min for each wash) to remove impurities that had filtered together with nuclei. The nuclei pellet was resuspended in an N2 buffer and then proceeded to quality control before immediate HS-AFM scanning or storage at −80 °C. For better preservation, the cryoprotectant Bambanker was used. The results of quality control are presented in Figure 2. Isolated nuclei were checked via the fluorescent cell imager (bright-field) (Figure 2a,b) and confocal microscopy (Figure 2c). Our purification method displayed clear visibility of nuclear surface markers such as nuclear envelopes (NE) and nuclear pore complexes (NPCs). Sample purity is important for HS-AFM imaging. If washing was inadequate (Figure 2a), these impurities could be removed using sucrose gradient density centrifugation (Figure 2b) without severely affecting the integrity of nuclei (Supplementary Figure S2).

### 3.2. Nanofabrication of Cantilevers Using Electron-Beam Deposition (EBD)

At high frequencies (400 kHz–1.2 MHz), as a cantilever gets closer to the sample surface, it compresses a tiny layer of water between the two surfaces. This leads to the dampening of the oscillation and reduces the sensitivity of detecting interactions between the tip and the sample. When the length of the tip exceeds around 2 μm, the impact of the squeezing effect becomes insignificant for a cantilever that is inclined at an angle of 10–20° from the surface of the substrate [69]. Insufficient length can be addressed by utilizing electron-beam deposition (EBD) to build an extra tip onto the existing one. Using a cantilever, we have enhanced a standard process for an EBD tip, specifically designed for obtaining nano-level pictures in a liquid environment.

**Figure 3:**
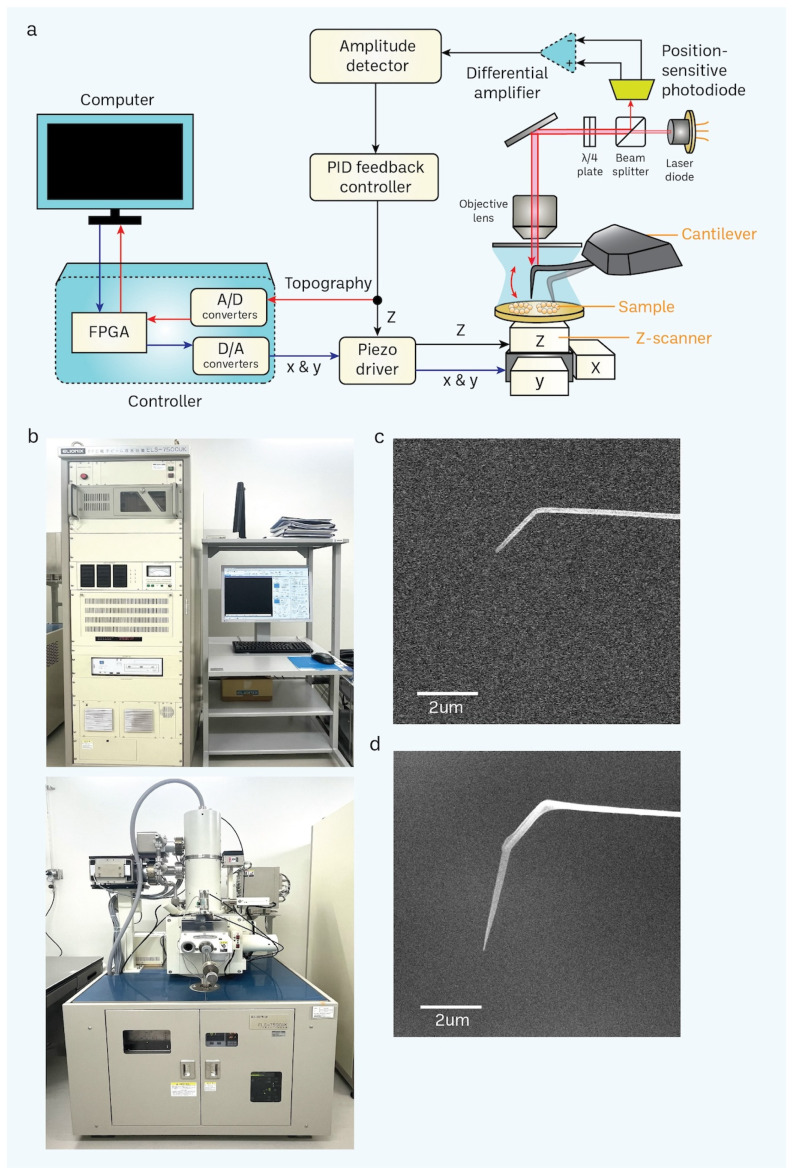
Basic principle of HS-AFM and setup of a scanning electron microscope (SEM). (**a**) A schematic diagram illustrates the principle of the HS-AFM system [69]. (**b**) SEM is used for nanofabrication of a cantilever through electron-beam deposition (EBD) to grow a carbon tip on the existing tip in a cantilever. (**c**,**d**) Difference between non-EBD (**c**) and EBD-ed (**d**) HS-AFM cantilevers are shown in the electron micrograph. EBD-ed cantilevers commonly have a length and radius of approximately 3 μm and 8 nm, respectively (scale bar, 2 μm).

We fabricated a vertically developed, elongated, thin tip composed of amorphous carbon. This tip was grown on a flexible AFM cantilever, which had a rigidity of 100–200 pN/nm. This was accomplished by utilizing a tailored EBD approach with the aid of a scanning electron microscope (SEM). After applying the surface treatment via cantilever cleaning with UV/O_3_, we attached the cantilever to a specifically engineered cantilever holder and inserted it into the chamber of the SEM. We utilized a focused electron beam to target the cantilever tip, employing an acceleration voltage of 50.0 kV. The duration of irradiation varied between 1 and 3 min, depending on the desired length of the tip [20,72]. The length of the cantilever can be measured via SEM imaging (Figure 3c,d), in which its application relies on its length. For example, a shorter tip cantilever is sufficient for the nanoimaging of proteins. In contrast, a longer tip cantilever [77,78] is ideal for the nanoimaging of organelles such as nuclei [20,72] and exosomes [79,80,81].

Additionally, our approach permits the recurrent utilization of a cantilever. Presently, it is customary to employ a cantilever for around 3–5 irradiation. The tip had a length of around 3 μm and a radius of around 8 nanometers during the scanning process (Figure 3d). The extended and sharp cantilever/tip design enables the high-resolution imaging of cells while minimizing stressful contact with the nucleus surface while scanning utilizing tapping mode (with a free amplitude of about 2 nm and a frequency of around 900 kHz). Furthermore, we have included a large-scale, rapid piezo scanner with a maximum scanning area of 3 × 3 μm^2^.

Regarding sample deposition, the outermost layer of a mica disk (with a diameter of 1.0–2.0 mm and a thickness of less than 0.1 mm) that is attached to a sample stage (a glass rod with a diameter of 1.5–2.0 mm and a length of 2 mm) is removed using a Scotch tape. This is performed by pushing the tape onto the mica disk and then peeling it off. For effective imaging, it is crucial to follow this straightforward process. The presence of loosely packed layers or burrs near the edge area of a mica disk might hinder imaging by causing mechanical instability. A quality check prior to HS-AFM imaging of nuclei samples is crucial to avoid impurities and nuclei aggregates. Freeze–thaw cycles could significantly affect the nuclei integrity, and this effect could be minimized using a cryoprotectant, Bambanker, to protect the nuclei. Typically, an undiluted nuclei solution was loaded on a poly-L-lysin-treated mica substrate for about 30 min to immobilize nuclei. After that, the substrate was washed with a scanning buffer to remove free nuclei. To avoid the drying of the solution, it is important to cover the sample stage with a humid hood during the incubation process, especially since the sample volume is small. Neglecting to take precautions against drying frequently results in poor imaging outcomes.

### 3.3. Acquisition of Nanoscopic Nuclear Pore Complex Topology of Normal Mice Brain Nuclei Using HS-AFM

**Figure 4:**
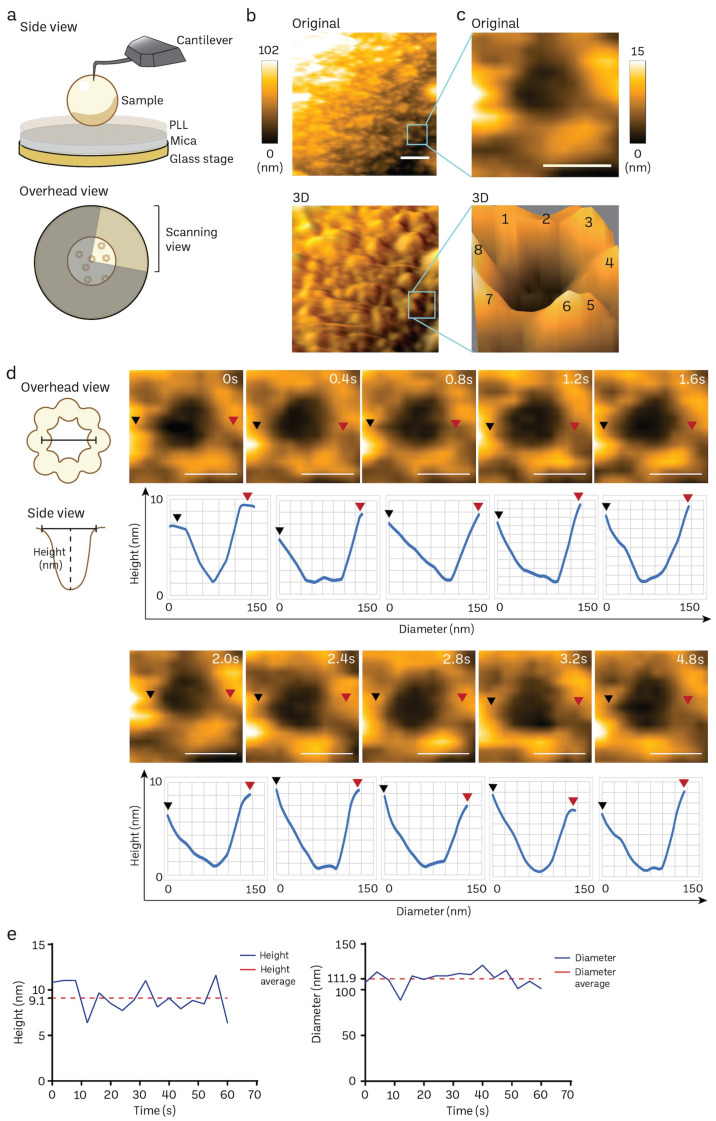
Nanoscopic elucidation of a native NPC structure of mouse brain nuclei using HS-AFM. (**a**) Schematic diagram of HS-AFM nanoimaging of a nucleus. (**b**,**c**) Cytoplasmic ring of a NPC on a nuclear envelope was captured using HS-AFM (scale bar, 400 nm), and the area was further zoomed to acquire a single NPC image (scale bar, 100 nm) together with its 3D image with an octameric ring-like structure (number 1–8) as shown in (**c**). NPC has a diameter of 115 ± 10 nm, and depth of 7 ± 2 nm (*n*:5). Data are presented as mean ± S.D. (**d**) A HS-AFM image sequence indicates the structural dynamics of a NPC filmed at a millisecond resolution (black arrow: starting point of measurement, red arrow: ending point of measurement, scale bar, 100 nm). (**e**) Real-time changes in height and inner diameter of a representative NPC measured using HS-AFM.

The poly-L-lysine (PLL)-modified mica is a suitable substrate to immobilize mice brain nuclei for HS-AFM nanoimaging, as delineated in Figure 4a. A solitary frame extracted from a HS-AFM film capturing the cytosol-facing outer nuclear membrane, which contains only a small number of nuclear pore complexes (NPCs), is shown in Figure 4b (also in Supplementary Video S1). We frequently observed individual NPCs exhibiting an octameric ring-like structure oriented towards the cytoplasm (Figure 4b,c). The average depth of the pore was approximately 7 ± 2 nm, while the average outer diameter measured 115 ± 10 nm (n:5). In line with prior findings in cells and organoids [20,72], we also noticed a significant variation in the openings of NPCs. We did not find significant differences in the NPC diameter and depth between normal mice and those nuclei isolated from cancer cell lines or cancer organoids. The high temporal resolution of HS-AFM (400 ms/frame) allowed us to acquire the dynamic movement of a cytoplasmic ring of the NPC, as demonstrated in Figure 4d. The graph underneath each sequential image exhibits the dynamic spatial conformations characterized by extension or retraction (Figure 4d). Additionally, the real-time fluctuation of height and outer diameter of the NPC can also be measured at a millisecond resolution (Figure 4e). These measurements are consistent with previous observations of NPCs in other mammalian cells using conventional AFM studies [20,72]. Collectively, our findings suggest that HS-AFM has the potential to capture the inherent spatial and temporal patterns of NPCs in mice brain tissues.

### 3.4. Transient Rapid Conformational Dynamics of FG-NUP Complexes Inside the Native Mice Brain Nuclear Pores

**Figure 5:**
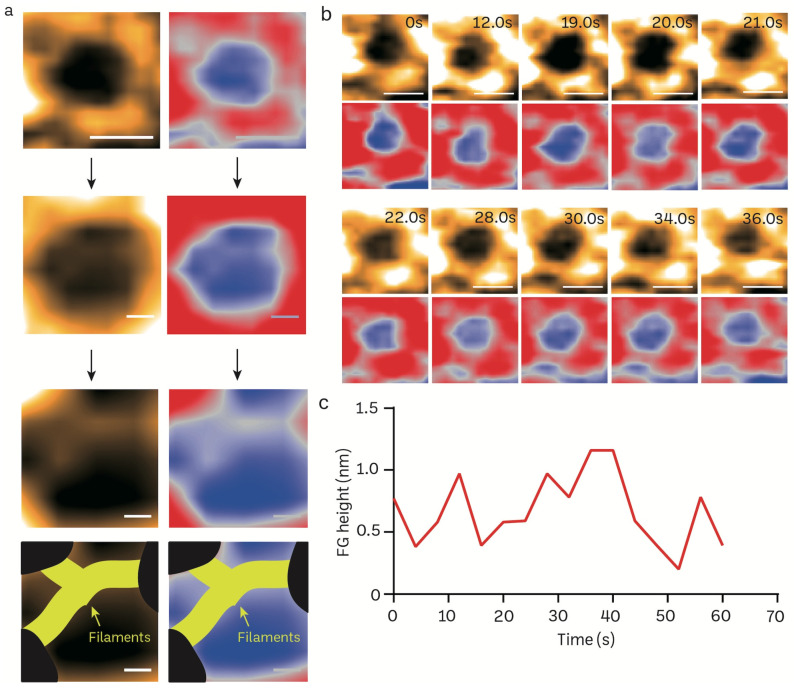
Structural dynamics of FG filaments in the central channel of a mice brain NPC visualized in real-time using HS-AFM. (**a**) Nanoscopic topology of central channel FG filaments (scale bar, upper: 100 nm; middle: 25 nm, bottom: 15 nm). (**b**) HS-AFM image sequence illustrates the dynamic properties of FG filaments. (**c**) A line graph depicts the real-time height change of an FG filament.

FG filaments in NPCs selectively regulate the influx and efflux of biomolecules between the nucleus and cytoplasm. The high spatiotemporal feature of HS-AFM allows us to capture the FG dynamics in a real-time manner. We reduced the scanning area (215 *×* 215 nm) to focus on the NPC orifice and then scanned it at 2.5 frames per second (Figure 5a). The enlarged pore demonstrates the filamentous FG structure that resides inside the pore. The inner nuclear central channel exhibited sequential motion variations, most likely due to the presence of FG-Nups, during a time period of 400 ms for each frame (Figure 5b; Supplementary Video S2). The FG-Nups that were stretched and retracted exhibited a structure resembling a broken spider web, as shown in Figure 5b. The FG-Nup filaments interacted in a very dynamic and reversible behavior. This interaction could form the complicated entanglement of the cobweb, or single filaments can overlap and twist over each other. Under the millisecond temporal resolution, we were able to measure the real-time height change of FG filaments using HS-AFM (Figure 5c).

## 4. Discussion

Isolation of cells from tissues or organs, especially clinical samples, is now a routine workflow for medical science research. Cellular nuclei provide important biological information for scientists and clinicians to study human diseases. Commonly, these biological samples are chemically fixed using formalin or rapid freezing with liquid nitrogen before proceeding to downstream experiments. Such harsh preparation could severely destroy the biological activities of these samples. Given that traditional nuclei isolation is a customary technique employed in molecular and cellular biology, we originally employed established techniques that involve tissue grinding using liquid nitrogen and fresh tissue chopping. By employing these techniques previously, we successfully retrieved an ample amount of nuclei [20,72] through the laborious process of sucrose density gradient centrifugation (Supplementary Table S1) [6,75]. Therefore, we established the rapid strainer microfiltration (RSM) protocol to isolate nuclei from tissues while minimally maintaining their biological activities. Our methodology significantly reduces the processing time from 6–8 h to 2.5 h (Supplementary Table S1). Subsequently, we employ real-time visualization and filming techniques to observe and record the behavior of nuclear pores at the nanoscale and millisecond levels.

Like other non-ionic detergents such as Triton X-100 and 2% digitonin, a low concentration of NP-40 (approximately 0.1–0.2%) is employed to permeabilize the cell membrane [82,83], allowing membrane rupture under mechanical stress. In our experiments, a mere 0.1% of NP-40 was applied in a short time. In certain cases, higher concentrations of NP-40, such as 0.3%, may be required for pure nuclei isolation, as observed in HeLa cells [82,83]. Although these mild detergents are well-known compounds for homogenization, we must be cautious when determining if the isolated nuclei are intact or semi-intact. To further assess nuclei integrity, additional NPC markers such as TPR, Nup88, RanBP2, and RanGAP1 were used for immunostaining (Supplementary Figure S2), indicating that the RSM method did not significantly alter the NPC gross morphology (cytoplasmic ring, cytoplasmic filaments, and central channel). In the near future, fluorescently tagged dextran or other transport assays will be used to better examine the intactness and integrity of the NPC in vitro.

NPCs enable the transport of certain macromolecules between the nucleus and cytoplasm while blocking the exchange of undesired substances. The large size and transmembrane properties make NPCs complex objects for study, especially when examining their structure. Through extensive and detailed investigations conducted over a prolonged period, full knowledge of the structure and positioning of the components of the NPC in the scaffold domain has been obtained. Nevertheless, the high-resolution images obtained from pure nuclear envelopes or in situ have traditionally been deficient in the essential FG-Nups, particularly those derived from yeast, *Xenopus*, and mammalian cells.

In this study, we demonstrate the isolation of nuclei using strainers with specific pore sizes and the subsequent, direct continuation of HS-AFM imaging of nuclei isolated from mice brains. The freshly isolated nuclei retained their native topologies because no chemical fixation was given, allowing us to determine the alteration of NPC topology caused by diseases such as cancers and neurodegenerative diseases. Moreover, the working environment for this protocol is helpful in preserving NPC biological functions, especially the activity of FG filaments that are responsible for biomolecular transport. The advantage of HS-AFM spatiotemporal resolution could enable us to study both structural and dynamic properties of FG filaments, in which their properties could be actively altered by gene expression or biochemical modification. For example, the overexpression of FG-Nups in cancer cells forms central plugs in the NPC [41], altering the permeability of the NPC that favors oncogenic signaling to support various hallmarks of cancer. Besides the topology of the NPC and dynamic properties of FG filaments, meaningful mechanical properties of the NPC, such as nuclear envelope or NPC stiffness and 3D-force mapping, can be acquired using conventional AFM on freshly isolated nuclei. These efforts are essential for the development of AFM-based nano-endoscopy that utilizes an AFM cantilever to visualize nuclei in living cells [84].

## 5. Conclusions

The use of sucrose gradient differential centrifugation has been widely employed to extract nuclei from cell lines and some tissues. However, isolating neuronal nuclei from fresh or frozen tissues is particularly challenging because of the presence of stromal tissues, making the process very labor-intensive. Using strainers, we can efficiently and precisely obtain the filtrate that is enriched with nuclei.

To summarize, the RSM method has greatly decreased the duration needed for isolating and purifying nuclei. This method also sets the stage for upcoming clinical research that utilizes patient tissues for HS-AFM nanoscopic imaging. This will enhance the accuracy of medical analysis and decision, enabling the transition from static images to dynamic videos at the nanoscale. In the future, it will enable the development of an artificial intelligence (AI) diagnosis model that utilizes a deep learning (DL) algorithm to diagnose various types of nuclei and NPCs. It is crucial to integrate the RSM protocol, nanoimaging technologies, and an AI-driven decision support system that utilizes clinical data and symptoms in the near future. This integration will aid physicians in quickly recognizing early phases of various symptoms and illnesses and providing prompt medication. Additionally, our nuclei isolation method yields high-quality nuclei suitable not only for nanoimaging but also for the separation and subsequent high-throughput single-cell molecular profiling, including snRNAseq and snATAC-Seq.

## Data Availability

The data used and analyzed in this study are available from the corresponding authors upon reasonable requests. The data are not publicly available due to institutional policy.

## Supplementary Materials

The following supporting information can be downloaded at: https://www.mdpi.com/article/10.3390/cells13030279/s1, Figure S1: Protocol for isolating and purifying nuclei from mouse tissue; Figure S2: Morphology of isolated nuclei observed by confocal microscope; Table S1: Comparison of nuclear isolation methods by sucrose gradient and current efficient isolation method; Video S1: Nanotopology of nuclear membrane and representative of a single NPC; Video S2: Nanotopology of a single NPC with FG-filament inside the NPC.
