# Supplementary material for: An Efficient Method for Isolating and Purifying Nuclei from Mice Brain for Single-Molecule Imaging Using High-Speed Atomic Force Microscopy
Source: Cells. 2024 Feb 2;13(3):279. doi: 10.3390/cells13030279 (PMC10855070; doi:10.3390/cells13030279)
Supplement: Supplementary file 1 [file cells-13-00279-s001.zip › 20240128 SI and Movie Captions Ver 3a.pdf]

Supplementary Materials

Figure S1-S2
Tables S1
Captions for Videos S1-S2

Other Supplementary Materials for this manuscript include:

Videos S1-S2

__Supplementary Figures__

Figure S1

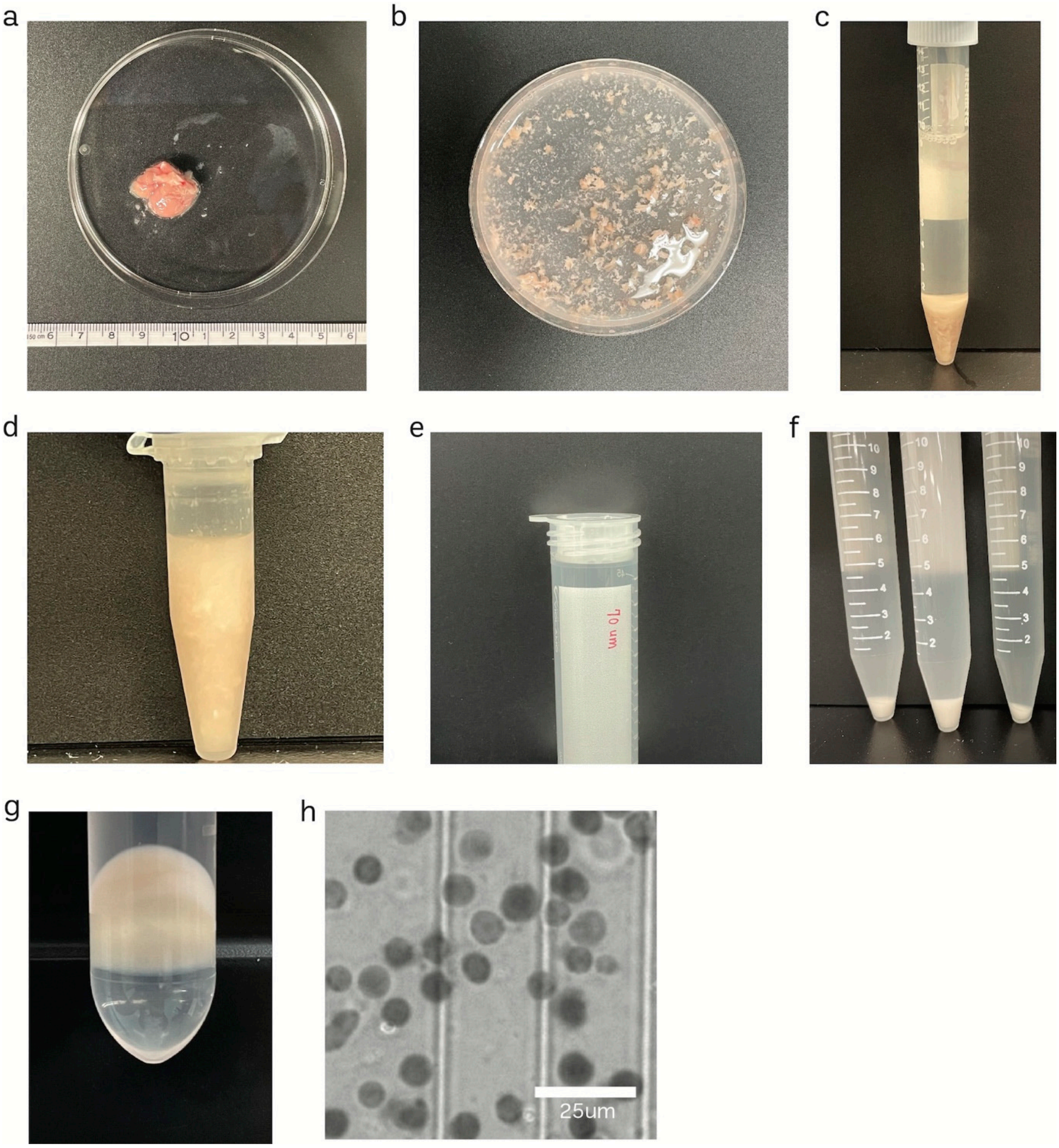

Protocol for isolating and purifying nuclei from mouse tissue.

(a) The mouse brain tissue samples underwent multiple washes using PBS and NIM1 buffer to eliminate blood and debris adhered to the tissue surface. (b, c) The mouse brain was partially sectioned into small fragments and then subjected to centrifugation at 300 g for 8 minutes. (d) The resulting pellet was finely ground with 12-15 pestle movements and subsequently placed

on ice for 10 minutes before passing through a 70-micrometer cell strainer. (e) After an 8-minute centrifugation at 300 rpm, the solid mass was combined with 13 mL of chilled phosphate-buffered saline (PBS) containing 1% bovine serum albumin (BSA) at a pH of 7.4. The supernatant underwent filtration through 70 um cell strainers until no liquid remained, followed by additional filtration through 40- and 20 um strainers. (f) The liquid was then centrifuged at a force of 300 g for 10 minutes. (g) The resulting pellet was mixed with an N3:N2 (1:2) buffer and layered on top of the N3 underlayer, undergoing ultracentrifugation at 17,000 g for 45 minutes. (h) The pellet was subsequently examined with trypan blue under a light microscope and finally resuspended in bambanker before storage at -80C.

Figure S2

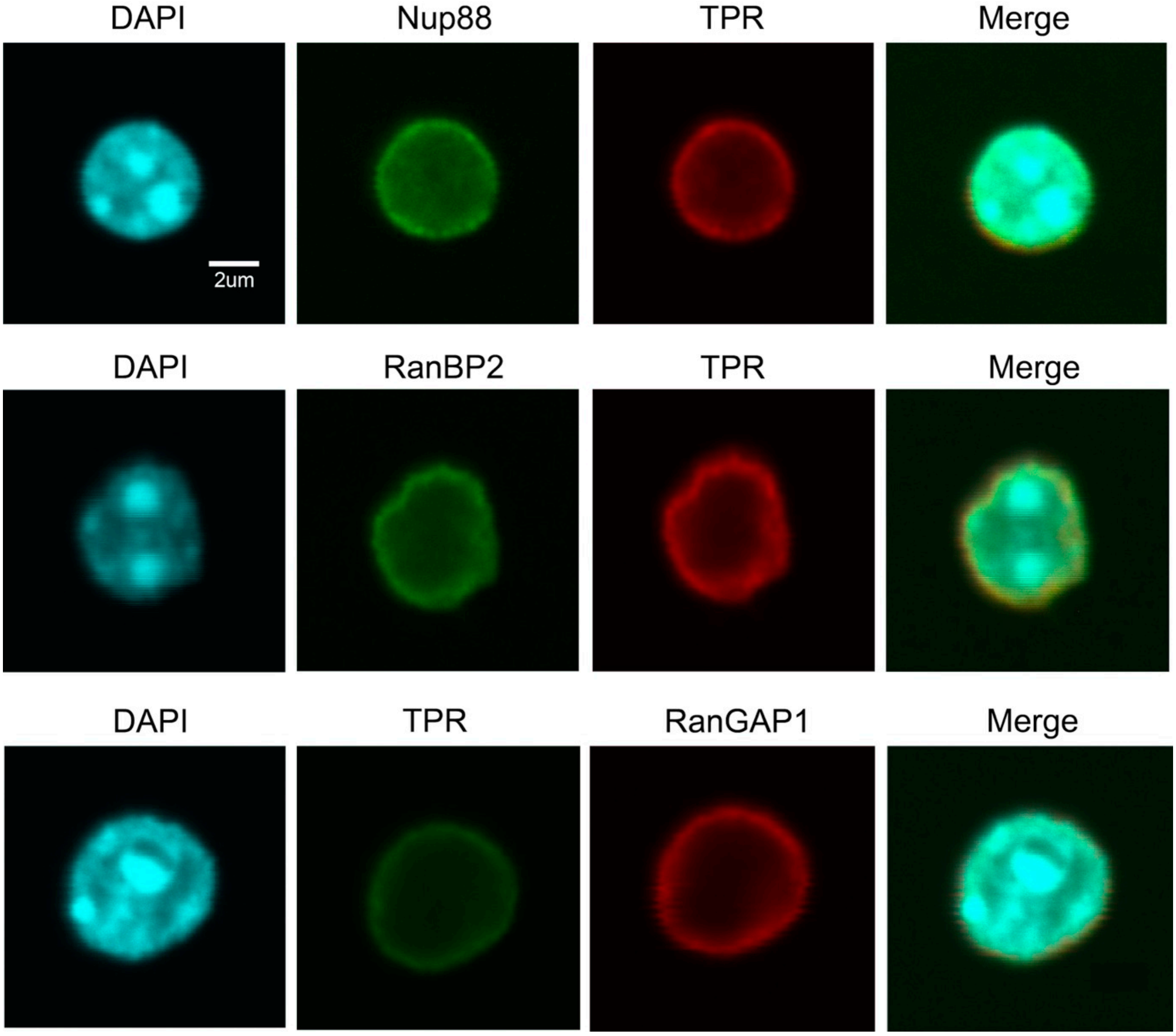

Morphology of isolated nuclei observed by confocal microscope.

Morphology and immunofluorescence analysis of isolated nuclei with indicated antibodies (TPR, Nup88, RanBP2, and RanGAP1) (scale bar: 2 μm).

Supplementary Tables

Table S1. Comparison of nuclear isolation methods by sucrose gradient and current efficient isolation method.

Sucrose gradient protocol		Strainer microfiltration protocol	
Methods	Estimated time	Methods	Estimated time
Buffer preparation	1 hour	Buffer preparation	30 minutes
↓ Cells collected by crapping in cold PBS containing PI (20 x 10cm dishes)	1 hour	↓ Brain tissue washing	5 minutes
↓ Centrifugation at 4°C, 15 minutes, 3,000g	15 minutes	↓ Brain tissue mincing	15 minutes
↓ Resuspend pellet in homogenization buffer (N1) and incubate	1 hour	↓ Centrifugation at 4°C, 8 minutes, 300g	8 minutes
↓ Rupture cells with homogenizer	30 minutes	↓ Resuspend tissue pellet in homogenization buffer (HB buffer)	
↓ Centrifugation at 4°C, 15 minutes, 2,200g	15 minutes	↓ Rupture cells with homogenizer and incubate 10 minutes	30 minutes
↓ Resuspend pellet in N2 buffer		↓ Centrifugation at 4°C, 5 minutes, 300rpm	5 minutes
↓ Centrifugation at 4°C, 15 minutes, 2,200g	15 minutes	↓ Strained the supernatant on 70-, 40-, and 20um strainer	30 minutes
↓ Prepare 0.5mL of N3 as underlayer in tube		↓ Centrifugation at 4°C, 10 minutes, 300g	10 minutes
↓ Resuspend pellet in 1.2mL of N3:N2 mixture (2:1)	5 minutes	↓ Resuspend pellet in bambanker and keep in -80°C	
↓ Slowly add the pellet mixture to N3 underlayer			
↓ Centrifuge for 45 minutes, 4°C, 17,000g	45 minutes		
↓ Resuspend pellet in N2 buffer (3 times)	45 minutes		
↓ Centrifugation at 4°C, 15 minutes, 2,200g			
↓ Resuspend pellet in N2 buffer and keep in -80°C			
TOTAL TIME	**5 hours 50 minutes**	TOTAL TIME	**2 hours 13 minutes**

Video Captions

Video S1: Nanotopology of nuclear membrane and representative of a single NPC.

Mouse nuclei were loaded on PLL-coated glass stage and scanned under a physiological buffer (50mM Tris-HCl, 150mM NaCl, pH 7.50). Nuclear membrane (a) was recorded at scanning speed of 7.5 frames/s and single NPC (b) was recorded at scanning speed 2.5 frames/s. Scanning area: (a) 2000 x 2000 nm with 120 x 120 pixels, (b) 215 x 215 nm with 120 x 120 pixels. Selected images of nuclear membrane are shown in Figure 4b, while images of single nuclei are shown in Figure 4c and d, and Figure 5a. All movies are merged into a single clip with playback speed of 60 fps (scale bar, a: 400nm, b: 43nm).

Video S2: Nanotopology of a single NPC with FG-filament inside the NPC.

The single NPC (a) and FG-filament (b) was recorded at scanning speed 2.5 frames/s. Scanning area: (a) 215 x 215 nm with 120 x 120 pixels, (b) 100 x 100 nm with 120 x120 pixels. Selected images are shown in Figure 5c. All movies are merged into a single clip with playback speed of 60 fps (scale bar, a:43 nm, b: 25nm).
